# Invasive Management of Vertebrobasilar Artery Stenosis and Occlusion: A Meta-Analysis on Efficacy and Safety Endpoints

**DOI:** 10.7759/cureus.24751

**Published:** 2022-05-05

**Authors:** Nso Nso, Mahmoud Nassar, Mia Trimingham, Yolanda Mbome, Anthony Lyonga Ngonge, Solomon O Badejoko, Shahzad Akbar, Atika Azhar, Sofia Lakhdar, Muhammad Ghallab, Laura M Guzman Perez, Vincent Rizzo, Most Sirajum Munira

**Affiliations:** 1 Internal Medicine, Icahn School of Medicine at Mount Sinai, New York City (NYC) Health and Hospitals/Queens, New York, USA; 2 Internal Medicine, American University of Antigua, College of Medicine, New York, USA; 3 Internal Medicine, Richmond University Medical Center, New York, USA; 4 Department of Medicine, The State University of New York (SUNY) Upstate University Medical Center, New York, USA; 5 Internal Medicine, St. Joseph’s Medical Center, Stockton, USA; 6 Internal Medicine, Kettering Medical Center, Dayton, USA; 7 Internal Medicine, Upstate University Hospital, Syracuse, USA; 8 Medicine, Icahn School of Medicine at Mount Sinai, New York City (NYC) Health and Hospitals/Queens, New York, USA; 9 Internal Medicine/Cardiology, Weill Cornell Medicine, New York, USA; 10 Cardiology, Icahn School of Medicine at Mount Sinai, New York City (NYC) Health and Hospitals/Queens, New York, USA

**Keywords:** safety and efficacy, stroke, posterior circulation stroke, vertebrobasilar ischemia, vertebrobasilar circulation

## Abstract

Vertebrobasilar angioplasty and stenting or mechanical thrombectomy (MT) using a stent retriever or suction thrombectomy are effective interventions in managing acute ischemic stroke caused by vertebrobasilar artery occlusion (VBAO). This study aims to investigate the safety and efficacy of self-expanding stents and balloon angioplasty in managing ischemic stroke. We reviewed the literature for relevant clinical trials and included those reporting the following primary outcomes: successful recanalization, favorable clinical outcome, and stenosis degree change. We included 24 studies (858 patients). In the subgroup analysis, participants were divided into three main subgroups based on the type of intervention: mechanical thrombectomy (MT), percutaneous transluminal angioplasty and stenting (PTAS), and MT+PTAS. Regarding overall mortality, the incidence was 34.5%, 9.9%, and 28.9% in the MT, PTAS, and MT+PTAS groups, respectively. The incidence of arterial dissection was 3.6% in the MT group, 3.1% in the PTAS group, and 16.7% in the MT+PTAS group. Incidence of distal embolization, MT, PTAS, and MT+PTAS groups had 3.4%, 5.8%, and 9.5% incidence rates, respectively. Favorable clinical outcomes were reported in 42.8% of subjects in the MT+PTAS group, 64.7% in the PTAS group, and 39.2% in the MT group. The incidence of intracranial hemorrhage was 5.2%, 4.5%, and 15.3% in the MT, PTAS, MT + PTAS groups, respectively. The incidence of successful recanalization was 85.3% in the MT group, 99.4% in the PTAS group, and 92.7% in the MT+PTAS group. Our analysis concludes that PTAS is the most effective intervention for VBAO and is associated with a lower rate of mortality compared to mechanical thrombectomy alone.

## Introduction and background

Stroke is the leading cause of death and disability worldwide [1]. Ischemic stroke accounts for 87% of cerebrovascular accidents (CVAs) [2]. The vertebrobasilar artery supplies the brain stem, cerebellum, occipital lobe, posterior temporal lobe, and thalamus [3]. VBAO represents about 20% of all ischemic strokes occurring in the posterior circulation [4-6]. The most common causes of vertebrobasilar artery occlusion are embolism, atherosclerosis, penetrating-small artery diseases, and arterial dissection [5-8].

Many cases of VBAO are undiagnosed or misdiagnosed [9]. This is likely because the most common initial symptoms are nonspecific, including but not limited to vertigo, dizziness, vomiting, and head or neck pain [9,10]. Further, CT or MR angiography reveals stenosis or occlusion of the affected artery in about 25% of posterior circulation strokes [11,12]. Currently, the standard care varies depending on the location of the occlusion. Due to the lack of data from randomized clinical trials, decisions regarding extradural occlusion are mostly dependent upon clinician judgment. In addition to risk factor modification, management options include single antiplatelet therapy and PTAS [13]. Unfortunately, antithrombotic agents and intravenous thrombolytics have yielded poor results in achieving recanalization of affected vasculature [14-18]. Several studies suggest that mechanical thrombectomy (MT) using devices such as stent retriever or suction thrombectomy is a safe and effective treatment for acute ischemic stroke caused by vertebrobasilar artery occlusion [19-21].

Vertebrobasilar angioplasty and stenting are effective options for patients with atherosclerotic vertebrobasilar disease [22]. There are various methods for achieving successful recanalization, including intra-arterial thrombolysis and percutaneous transluminal angioplasty (PTA) [23]. Although surgical management is a well-known and effective treatment option for intracranial vertebrobasilar atherosclerosis, there are high morbidity and mortality rates [24,25]. While the percutaneous management of vertebral artery occlusion was associated with lower morbidity than surgical repair [26], VBAO is generally associated with a relatively good prognosis. The only factor favoring better outcomes and prognosis is early recanalization of the occluded vessels [27-29].

A limited number of studies have been conducted regarding the safety and efficacy of self-expanding stents and balloon angioplasty in the management of ischemic stroke secondary to VBAO. Therefore, we conducted this systematic review and meta-analysis.

Methodology

We followed the criteria of preferred reporting items for systematic reviews and meta-analyses (PRISMA) in designing our systematic review and meta-analysis [30].

Literature Search

We searched the following databases for relevant articles published through November 2020: PubMed, Cochrane Central, Scopus, and Web of Science. We used the following keywords "basilar," "vertebral," "vertebra-basilar," "recanalization," "revascularization," "occlusion," "stenosis," "thrombosis," "stent," "thrombectomy," and "angioplasty." All authors screened the titles and abstracts of the obtained records independently according to the eligibility criteria, followed by full-text screening, and when there was a conflict about the inclusion decision, it was solved by discussion.

Eligibility Criteria

We included one-arm retrospective and prospective observational cohort studies of patients with basilar, vertebral, or vertebral-basilar artery occlusion who had undergone an invasive intervention: mechanical thrombectomy or angioplasty with or without a stent. Our primary outcomes were successful recanalization, favorable clinical outcome, and stenosis degree change. Secondary outcomes included mortality, postoperative complications, NIHSS (National Institute of Health Stroke Scale) score change, need for retreatment, and Modified Rankin Scale (MRS) score change.

Data Extraction

We independently extracted data related to patient characteristics, procedure-related complications, and outcomes. Patient characteristics included age, gender, presenting symptoms, comorbidities, and site of occlusion. Procedure outcomes were post-procedure successful recanalization rate, favorable recanalization at three months, post-procedure NIHSS change, post-procedure MRS change, and post-procedural stenosis change. Post-procedure-related complications were the need for retreatment, infarction, intracranial hemorrhage (ICH), stent embolism, re-occlusion, restenosis, artery dissection, distal emboli, transient ischemic attack, and stroke. Successful recanalization was defined as a Modified Treatment in Cerebral Ischemia (mTICI) score of 2B or 3 measured after procedure performance or technical success. A favorable outcome was defined as the MRS between (0-2) at three months of follow-up.

Quality Assessment

We assessed the quality of the included studies by the Newcastle Ottawa scale (NOS). The NOS contains three main domains: selection, comparability, and ascertainment of the outcome. It is based on reviewer judgment by marking stars on specific items under each domain if matched in the included studies. A high number of total stars represents good quality.

Meta-Analysis

We calculated the qualitative outcomes by pooling each study's proportions by the untransformed proportion equation, and the pooled proportion was presented with a 95% confidence interval (95% CI). Regarding quantitative outcomes, we calculated the change in MRS and NIHSS according to the Cochrane Handbook for Systematic Reviews of Interventions [31], then a meta-analysis was performed by pooling mean change values of each study using the inverse-variance method, and the pooled mean change value was presented with 95% CI. Results were considered significant when the p-value was less than 0.05. We used OpenMeta [Analyst] (an open source software available at http://www.cebm.brown.edu/openmeta/) to perform this meta-analysis.

Heterogeneity

We used the random-effects model as the existent difference between studies on patient characteristics, severity, site of occlusion, and various procedures. We tested heterogeneity by the chi-square test. Outcomes were considered homogenous if the P-value was more than 0.1 and I2 was less than 50%. In the case of heterogeneous outcomes, we performed a sensitivity test and searched for the cause of heterogeneity.

## Review

Search results

**Figure 1:**
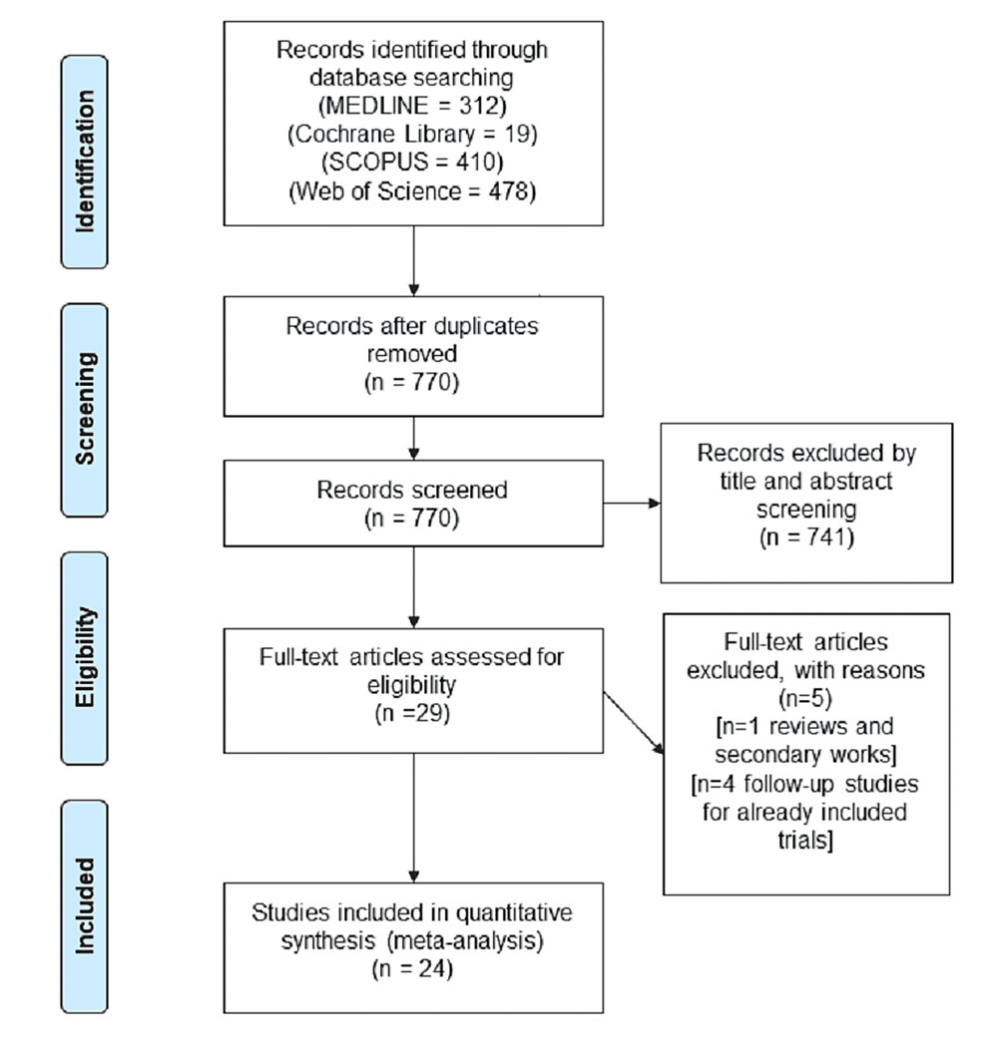
PRISMA flow diagram of our literature search PRISMA: Preferred reporting items for systematic reviews and meta-analyses

Our search of four databases revealed 1219 results. By Endnote software, 449 studies were excluded due to duplication. We performed title and abstract screening for the remaining 770 results. Study outcomes that were irrelevant to our study, posthoc analyses, non-English language studies, review articles, conference abstracts, editorials, or individual case reports were excluded. Only 29 of them were eligible for full-text screening. After the full-text screening, we included 24 studies according to our inclusion criteria. Twenty-two studies were retrospective cohort studies, and the two studies were prospective cohorts. We searched all references included in each study manually, but no further records were added to the included studies. We excluded five studies in the full-text screening for reasons including stenting of other cranial vessels. The PRISMA flow diagram is shown in Figure 1.

Characteristics of included studies

The 22 cohort studies recruited 864 participants. The earliest of them was published in 1999 and the latest in 2019. Six studies were conducted in China, four in Germany, three in the USA, three in Australia, two in Japan, and one study in each of these countries: the UK, Turkey, Korea, and Spain. According to the type of treatment, three subgroups were defined: Percutaneous transluminal angioplasty with or without stenting (PTAS), Mechanical thrombectomy (MT), and combination PTAS+MT. A detailed summary of the characteristics of both included studies and the participants is illustrated in Table 1.

**Table 1:** Baseline summary of included studies Abbreviations: BA, Basilar artery; BMS, Balloon mounted stenting; BPDPS, Balloon pre-dilation plus self-expanding stenting; MT, Medical treatment; MTh, Mechanical thrombectomy; PTA, percutaneous transluminal angioplasty; PTAS, Percutaneous transluminal angioplasty, and stenting; S, Stenting; TIA, Transient ischemic attack; VA, Vertebral artery; VBS, Vertebrobasilar system. Barakate et al. 2001 [32]; Broussalis et al. 2011 [33]; Canyigit et al. 2007 [43]; Chastain et al. 1999 [44]; Djurdjevic et al. 2019 [34]; Eberhardt et al. 2006 [45]; Fiorella et al. 2007 [35]; Gao et al. 2015 [36]; Gao et al. 2018 [46]; Huo et al. 2016 [37]; Karameshev et al. 2010 [58]; Kim et al. 2015 [27]; Kowoll et al. 2013 [47]; Levy et al. 2001 [48]; Mohlenbruch et al. 2014 [38]; Parkhutik et al. 2010 [22]; Quan et al. 2019 [39]; Shore et al. 2019 [49]; Tsutsumi et al. 2007 [40]; Wajima et al. 2017 [13]; Wang et al. 2015 [42]; Weber et al. 2005 [41]; Zhang et al. 2019 [50]; Wehman et al. 2004 [59]

Author and Year	Country	N	Study design	Male	Age: Mean (SD); range years	Baseline NIHSS score	Clinical presentation	Site of occlusion	Follow-up period: mean (SD); range months
Barakate et al. 2001	Australia	PTAS= 11	Retrospective cohort	91%	66; 56-75	.	Dysarthria, vertigo, and visual disturbance	73% VA and 27% BA	3
Broussalis et al. 2011	Austria	PTAS= 22	Retrospective cohort	64%	51-82	0-12	Cerebellar infarction, and brainstem infarction	VA	12
Canyigit et al. 2007	Turkey	PTAS= 35	Retrospective cohort	87.50%	60.3; 32-76	.	Diplopia, dysarthria, and vertigo	VA	6
Chastain et al. 1999	US	PTAS= 50	Retrospective cohort	74%	62.6 (9.1); 23-86	.	TIA and stroke (94%) and asymptomatic (6%)	VA	25 (10)
Djurdjevic et al. 2019	UK	PTAS= 24	Retrospective cohort	79.10%	68; 33-85	07-Nov	TIA and stroke	VA	18
		PTAS= 6		83.30%	67; 59-73			BA	
Eberhardt et al. 2006	Germany	PTAS= 20	Retrospective cohort	.	48-77	.	TIA and stroke	80% VA and 20% BA	Jun-36
Fiorella et al. 2007	US	PTAS= 44	Retrospective cohort	79.50%	64.8	.	Ischemic symptoms	VBS	12
Gao et al. 2015	China	MTh+PTAS= 30	Retrospective cohort	84.60%	56 (6.2); 19-34	6; 19-34	Ischemic symptoms	76.9% BA and 13% VA	3
Gao et al. 2018	China	PTAS= 14	Retrospective cohort	100%	43-64	.	dizziness,	VB	3
							nausea, and vertigo		
Huo et al. 2016	China	PTA = 36	Prospective cohort	83.30%	58.6 (8.1)	25.5	Ischemic symptoms	BA	3
Karameshev et al. 2010	Switzerland	MT = 29	Prospective cohort	59%	68 (8)	1	TIA and stroke	VA	33.6
		S = 10		80%	60 (13)	3.5			
Kim et al. 2015	Korea	PTA = 14	Prospective cohort	43%	49-83	19.5	Stroke	VBS	3
Kowoll et al. 2013	Germany	PTA = 12	Retrospective cohort	66%	68; 45-83	14.3	Cerebellar infarction	50% VA and 50% BA	12
Levy et al. 2001	US	PTAS = 11	Retrospective cohort	100%	43-77	.	TIA and stroke	VBS	36
Mohlenbruch et al. 2014	Germany	PTAS = 24	Retrospective cohort	70%	70; 33-83	24	Ischemic symptoms	BA	3
Parkhutik et al. 2010	Spain	PTAS = 28	Prospective cohort	75%	64 (9)	.	Symptomatic 50% and asymptomatic 50%	VA	32 (24)
Quan et al. 2019	China	MTh = 89	Retrospective cohort	75%	62; 52-69	17; 14–21	Ischemic symptoms	VBS	3
		MTh+PTA = 43							
		PTAS = 27							
Shore et al. 2019	Australia	MTh+PTAS = 28	Retrospective cohort	46%	65.2; 20-89	.	Weakness or sensory change 68%	BA	.
Tsutsumi et al. 2007	Japan	PTAS= 12	Retrospective cohort	66%	58-81	.	Infarction and ischemic symptoms	VA	13
Wajima et al. 2017	Japan	PTAS= 8	Retrospective cohort	75%	69 (11); 54-80	.	Vertigo, nausea, and dysarthria	VBS	10.3
Wang et al. 2015	China	PTAS= 88	Retrospective cohort	75%	62.6 (10.1); 41-82	.	TIA 61%	VA	12
Weber et al. 2005	Germany	PTAS= 21	Retrospective cohort	71%	67 (10)	.	Ischemic symptoms	VBS	9
Zhang et al. 2019	China	BMS = 98	Retrospective cohort	74.50%	60.49 (8.29)	.	TIA and stroke	VBS	12
		BPDS = 69		84.10%	58.67 (9.52)	.			
Wehman et al. 2004	US	PTAS= 55	Retrospective cohort	.-	.-	.	Ischemic symptoms	VA	12

Comorbidities

Eighteen studies reported the presence of baseline comorbidities with the potential to influence the success of intervention or contribute to further complications. These comorbidities included hyperlipidemia/ hypercholesterolemia, hypertension, diabetes mellitus, coronary artery disease, peripheral vascular disease, smoking, and alcohol consumption. This is summarized in Table 2.

**Table 2:** Comorbidity TIA: Transient Ischemic Attack; CAD: Coronary Artery Disease; PVD: Peripheral Vascular Disease; DM: Diabetes Mellitus. Barakate et al. 2001 [32]; Broussalis et al. 2011 [33]; Canyigit et al. 2007 [43]; Chastain et al. 1999 [44]; Gao et al. 2018 [46]; Huo et al. 2016 [37]; Kim et al. 2015 [27]; Kowoll et al. 2013 [47]; Levy et al. 2001 [48]; Mohlenbruch et al. 2014 [38]; Parkhutik et al. 2010 [22]; Quan et al. 2019 [39]; Shore et al. 2018 [49]; Tsutsumi et al. 2006 [40]; Wajima et al. 2017 [13]; Wang et al. 2015 [42]; Weber et al. 2005 [41]; Wehman et al. 2004 [59]; Zhang et al. 2019 [50]

Study ID	Hypertension		CAD, coronary artery disease		PVD, peripheral vascular disease		tobacco / active smoker		DM, diabetes mellitus;			hyperlipidemia/hypercholesterolemia		atrial fibrillation		history of previous TIA or stroke		Alcohol	
	event	total	event	total	event	total	event	total		event	total	event	total	event	total	event	total	event	total
Barakate et al. 2001	14	22	10	22	0	22	4	22		4	22	16	22	4	22				
Broussalis et al. 2011																			
Canyigit et al. 2007	9	16	7	16			9	16		5	16	1	16						
Chastain et al. 1999	38	50	38	50	17	50	21	50		12	50	24	50	1	50				
Gao et al. 2018			2	14			8	14		4	14								
Huo et al. 2016	25	36					28	36		12	36			2	36			23	36
Kim et al. 2015	11	14					1	14		4	12	3	12	8	14	3	14		
Kowoll et al. 2013	11	12	8	12						3	12			5	12	3	12		
Levy et al. 2001	7	11	5	11	5	11	5	11		1	11	3	11						
Mohlenbruch et al. 2014	15	24	5	24			6	24		6	24	12	24	8	24	8	24		
Parkhutik et al. 2010	10	16	9	16	2	16	5	16		8	16	8	16			Stroke = 13	16	4	16
																TIA = 3			
																all = 16			
	7	13	4	13	2	13	6	13		3	23	7	13			Stroke = 9	13	4	13
																TIA = 3			
																all = 12			
	17	53	13	29	4	29	11	29		11	29	15	29			Stroke = 22	29	8	29
																TIA = 6			
																all = 28			
Quan et al. 2019	59	159								44	159	31	159	44	159				
Shore et al. 2018	19	28			6	28	4	28		8	28	15	28	7	28	5	28		
Tsutsumi et al. 2006																			
Wajima et al. 2017	8	8								2	8	3	8						
Wang et al. 2015	68	88	22	88						46	88	44	88			stroke =20	88		
Weber et al. 2005																			
Wehman et al. 2004																			
Zhang et al. 2019	80	98					23	98			33	41				Sroke = 49	98		
																TIA = 49			
																all = 98			
	54	69					14	69			25	31				Sroke = 30	69		
																TIA = 39			
																all = 69			

Risk of bias assessment

**Figure 2:**
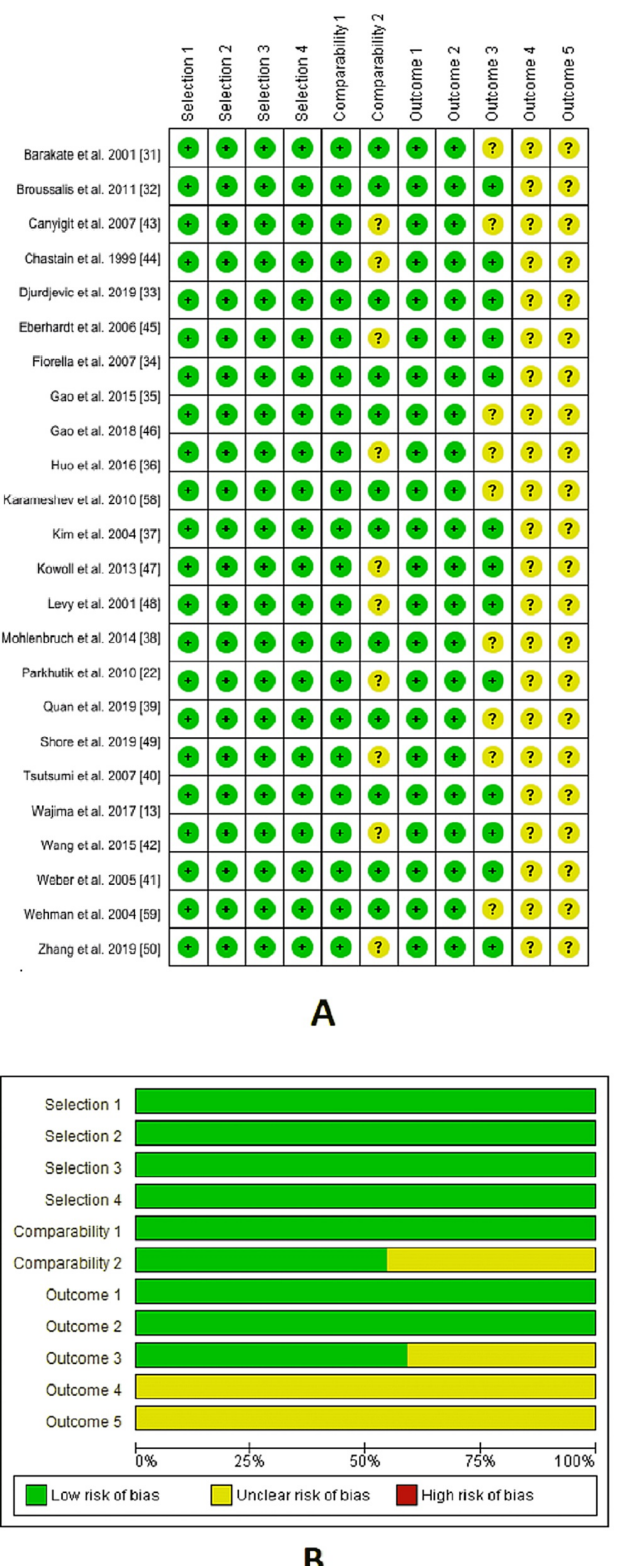
A summary and a graph showing the risk of bias in the included studies

The quality of the included studies ranged from moderate to high, as shown in the risk of bias graph, and summary (Figure 2).

Selection: Regarding the representativeness of the exposed cohort, all studies are of low risk of bias. The same was found to be true of the non-exposed cohort. Concerning the ascertainment of exposure, the presence of surgical records and follow-up interviews confer a low risk of bias. Finally, regarding the demonstration that the outcome of interest was not present at the start of the study, all studies are of low risk of bias.

Comparability: There was comprehensive matching for all plausible prognostic variables in 12 studies [31-42], so we considered them as low risk of bias. However, this item was not mentioned clearly in the remaining 10 studies [13,22,43-50], so we considered them of unclear risk of bias.

Outcome: Regarding confidence in assessing outcomes, all studies were of low risk of bias as an independent blind assessment was conducted. As for follow-up, all studies are of low risk of bias. Regarding the adequacy of cohorts' follow-up, 13 studies [13,22,32-34,37,40,42,44,45,47,48,50] reported adequate details suggesting no missing data or the missed data is not enough to have a significant impact on the intervention. Therefore, they were considered at low risk of bias. However, nine studies [31,35,36,38,39,41,43,46,49] did not report enough data about this outcome; thus, they were put at unclear risk of bias.

Analysis of outcomes

Primary Endpoints

**Figure 3:**
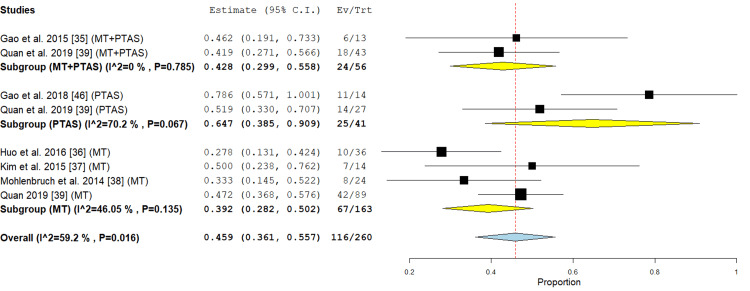
Forest plot for the analysis of favorable outcomes after three months PTAS: percutaneous transluminal angioplasty and stenting, MT: mechanical thrombectomy.

Favorable clinical outcome was defined as an MRS score ≤2 at three months and/or improvement of ≥10 or ≤6 points in the NIHSS score. The incidence rate was 64.7% in the PTAS subgroup as reported by two studies [39,46], (95% CI {38.5%, 90.9%}). The analysis showed marked heterogeneity (p = 0.067; I² = 70.2 %). Regarding the MT subgroup, four studies reported this outcome [36-39], with incidence of 39.2% (95% CI {28.2%, 50.2%}). The analysis showed no significant heterogeneity (P= 0.135; I² = 46.05 %) In the PTAS+MT subgroup, two studies [35,39] reported this outcome, with an incidence of 42.8% (95% CI {29.9%, 55.8%}), the analysis showed no significant heterogeneity (P= .785; I² = 0 %) (Figure 3).

**Figure 4:**
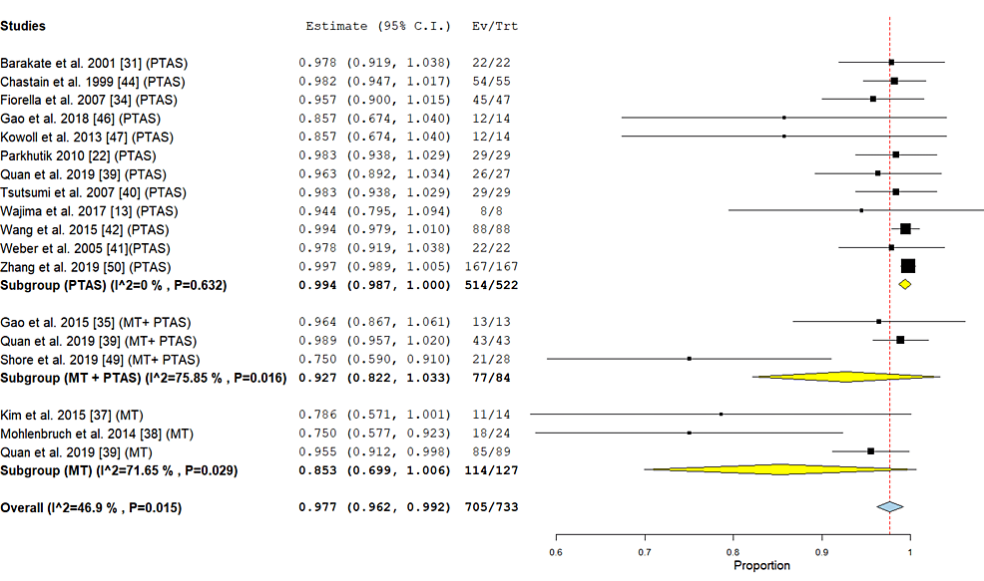
Forest plot for the analysis of successful recanalization PTAS: Percutaneous transluminal angioplasty, and stenting, MT: mechanical thrombectomy.

Successful recanalization: The PTAS subgroup included 12 studies [13, 22, 32, 34, 39-42, 44, 46, 47, 50] reporting this outcome, the incidence rate was 99.4% (95% {98.7%, 100%}), and the analysis of this subgroup was homogenous (P= 0.632; I² = 0 %). The MT subgroup included three studies [37-39], the incidence rate was 85.3% (95% CI {69.9%, 100%}), the analysis was heterogenous (P= 0.029; I² = 71.65 %). In the PTAS+ MT subgroup three studies [35,39,49] reported this outcome, the incidence rate was 92.7% (95% CI {82.2%, 100%}), the analysis was heterogeneous (P= 0.016; I² = 75.85 %) (Figure 4).

**Figure 5:**
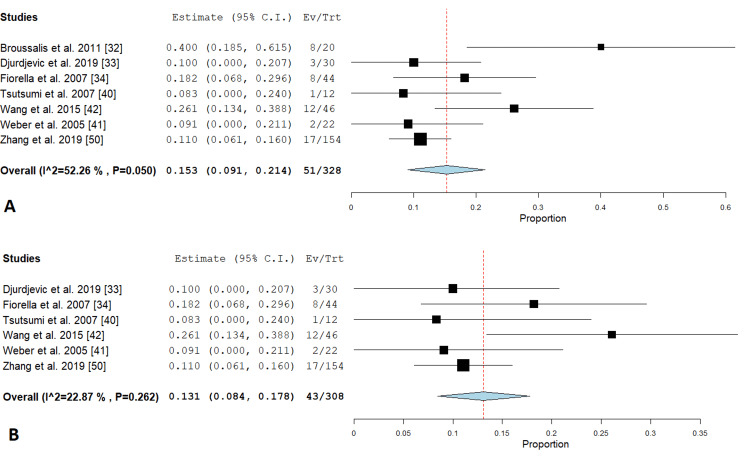
Forest plots for the analysis of restenosis (A) shows a forest plot for the analysis of restenosis, and (B) shows a forest plot for the analysis of restenosis after the exclusion of one study.

Restenosis: This outcome was illustrated in seven studies in the PTAS subgroup [32-34,40-42,50]. Pooled estimate showed significant liability for restenosis (0.153 {0.091, 0.214}), (P value < 0.001). Pooled analysis was heterogeneous (P = 0.050; I² = 52.264%) as shown in Figure 5A. We solved the heterogeneity by the exclusion of Broussalis et al. 2011 study [33] (P = 0.262; I² = 22.87 %). The pooled analysis after exclusion of the study also showed significant increase in restenosis incidence as a complication of PTAS (study estimate =0.131 {0.084, 0.178}) (P < 0. 01). Figure 5B illustrates the analysis after the exclusion of one study.

**Figure 6:**
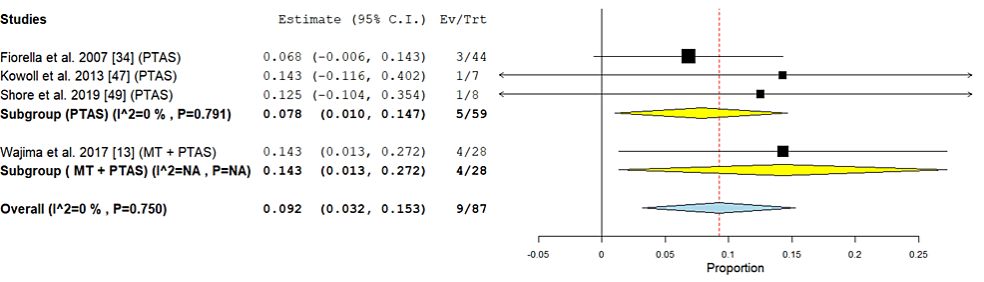
Forest plot for the analysis of re-occlusion PTAS: Percutaneous transluminal angioplasty, and stenting, MT: mechanical thrombectomy.

Re-occlusion: This outcome was represented in the PTAS subgroup in three studies [13,34,47] with an incidence rate of 7.8% (95% CI {1%, 14.7%}), the analysis showed no significant heterogeneity (P =0.791; I² = 0%) as shown in Figure 6.

**Figure 7:**
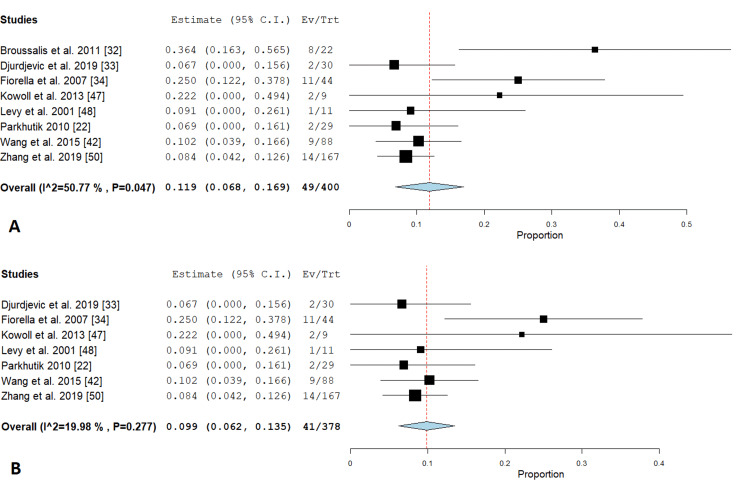
Forest plots for the analysis of stroke (A) shows a forest plot for the analysis of stroke, and (B) shows a forest plot for the analysis of stroke after excluding one study.

Stroke: This outcome was mentioned only in the PTAS subgroup in eight studies [22,31-34,42,47,48,50]. Pooled analysis showed that the incidence of stroke was 11.9% (95% CI {6.8%, 16.9%}); the analysis was heterogeneous (P= 0.047; I² = 50.77 %) as shown in Figure 7A. We solved the heterogeneity by the exclusion of Broussalis et al. 2011 study [33] (P = 0.277; I² = 19.98 %). Figure 7B illustrates the analysis after the exclusion of one study.

**Figure 8:**
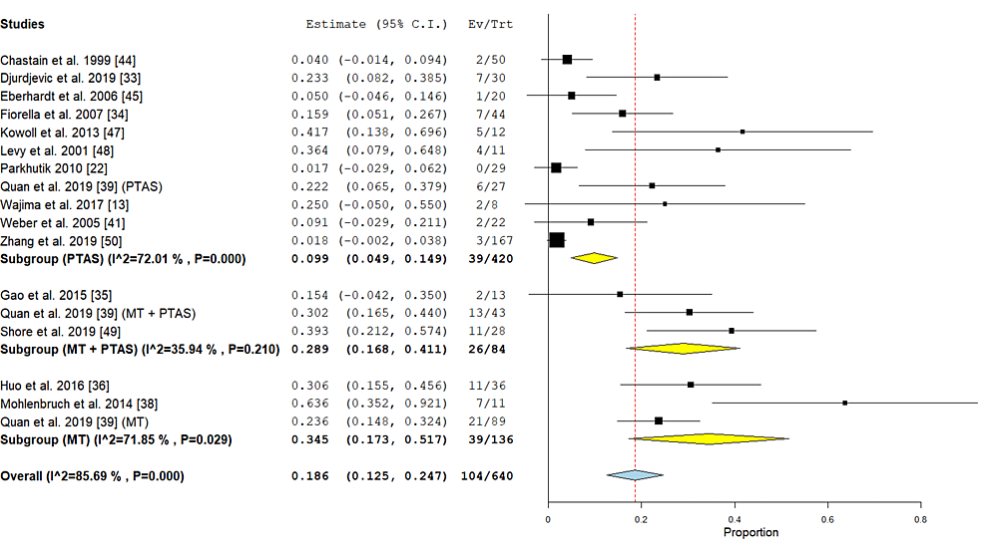
Forest plot for the analysis of mortality PTAS: percutaneous transluminal angioplasty and stenting, MT: mechanical thrombectomy.

Mortality: Eleven studies in the PTAS subgroup [13,22,33,34,39,41,44,45,47,48,50] reported this outcome, the incidence rate was 9.9% (95% CI {4.9%, 14.9%}), the analysis was heterogeneous (P < 0.001; I² = 72.01 %). In the MT subgroup three studies were included [36,38,39] with an incidence rate of 34.5% (95% CI {17.3%, 51.7%}), the analysis was heterogeneous (P=0.029; I² = 71.85 %). In the PTAS+MT subgroup three studies [35,39,49] reported this outcome with an incidence rate of 28.9% (95% CI {16.8%, 41.1%}), (P value < 0.001) and the analysis showed no significant heterogeneity (P= 0.210; I² = 35.94%) (Figure 8).

Secondary Outcomes

**Figure 9:**
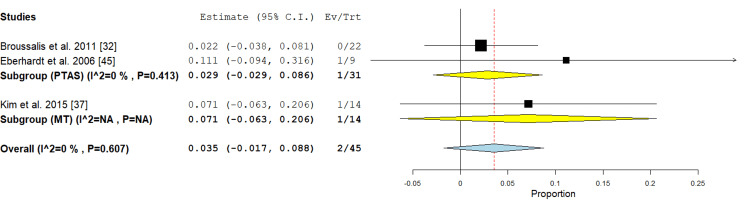
Forest plot for the analysis of embolism and thrombus PTAS: percutaneous transluminal angioplasty and stenting, MT: mechanical thrombectomy.

Secondary outcomes included other complications of the procedure and methods of evaluation after it. Embolism or thrombus: this outcome was represented in the PTAS subgroup in two studies [32,45]. In the MT subgroup, it was mentioned in one study only, so it could not be analyzed. Concerning PTAS subgroup, the incidence rate was 2.9% (95% CI {2.9%, 8.6%}), the analysis showed no significant heterogeneity (P =0.413; I² = 0%) as shown in Figure 9.

**Figure 10:**
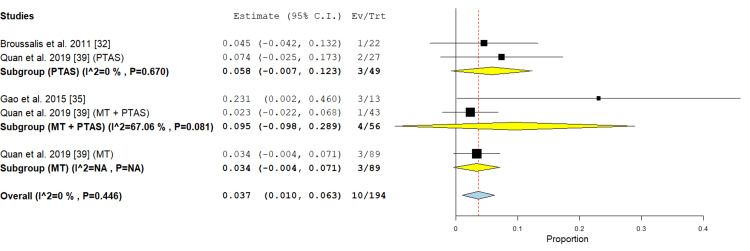
Forest plot for the analysis of distal emboli PTAS: percutaneous transluminal angioplasty and stenting, MT: mechanical thrombectomy

Distal emboli: In the PTAS subgroup, two studies [32,39] reported this outcome and the analysis revealed an incidence rate of 5.8% (95% CI {-0.007, 0.123}), the analysis of this subgroup showed no significant heterogeneity (P = 0.670; I² = 0 %). In the MT subgroup two studies were included, the incidence rate was 3.6% (95% CI {1%, 7.3%}), the analysis was homogenous (P= 0.597; I² = 0%). In the PTAS+MT subgroup two studies reported this outcome, the incidence of distal emboli reached 9.5% (95% CI {-0.098, 0.289}), the analysis was heterogenous (P=0.081; I² =67.06 %) (Figure 10).

**Figure 11:**
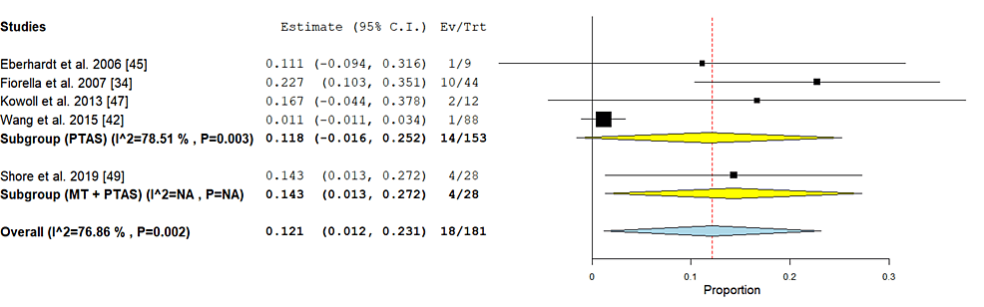
Forest plot for the analysis of infarction PTAS: percutaneous transluminal angioplasty and stenting, MT: mechanical thrombectomy.

Infarction: This outcome was represented in the PTAS subgroup by four studies [34,42,45,47]. The incidence of infarction was 11.8% (95% CI {-0.016, 0.252}). Pooled analysis was heterogenous (P = 0.003; I² = 78.51%), and this heterogeneity cannot be solved by study exclusion, as shown in Figure 11.

**Figure 12:**
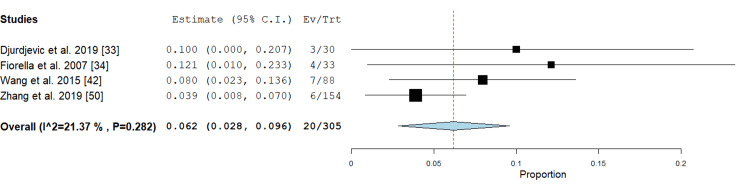
Forest plot for the analysis of the transient ischemic attack

Transient ischemic attack: This outcome was mentioned in four studies [33,34,42,50] in the PTAS subgroup, and the result of the analysis revealed an incidence of 6.2% (95% CI {2.8%, 9.6%}). Pooled analysis showed no significant heterogeneity (P= 0.282; I² = 21.37 %) (Figure 12).

**Figure 13:**
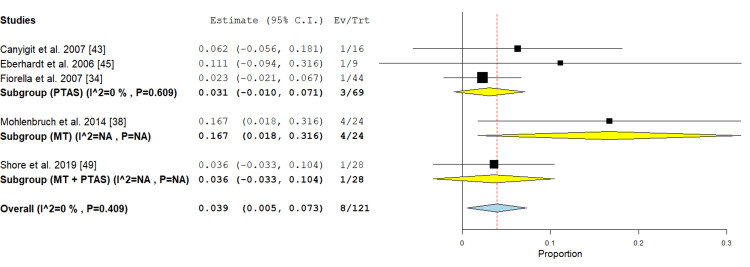
Forest plot for the analysis of artery dissection PTAS: percutaneous transluminal angioplasty and stenting, MT: mechanical thrombectomy

Artery dissection: This outcome was represented in the PTAS subgroup in three studies [34,43,45]. The incidence of artery dissection was 3.1% (95% {-0.010, 0.071}). Pooled analysis showed no significant heterogeneity (P =0.609; I² = 0%) as shown in Figure 13.

**Figure 14:**
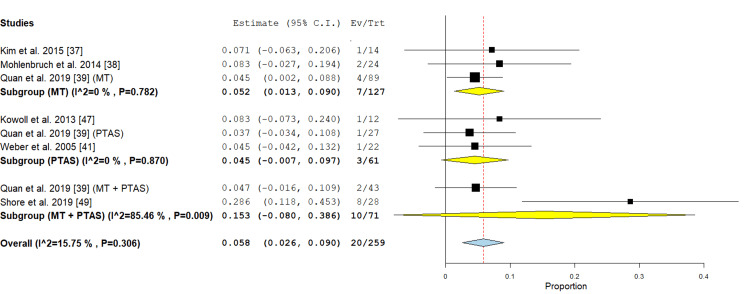
Forest plot for the analysis of ICH PTAS: percutaneous transluminal angioplasty and stenting, MT: mechanical thrombectomy

ICH: In the MT subgroup three studies were included [37-39]. The incidence of ICH was 5.2% (95% CI {1.3%, 9%}), the analysis of this subgroup showed no significant heterogeneity (P= 0.782; I² = 0 %). In the PTAS subgroup three studies [39,41,47] reported this outcome. The incidence was 4.5% (-0.007, 0.097), the analysis of this subgroup showed no significant heterogeneity (P= 0.87; I² = 0 %). In the PTAS+MT subgroup two studies [39,49] reported this outcome with an incidence of 15.3% (95% CI {-0.080, 0.386}), the analysis was heterogeneous (P=0.009; I² = 85.46 %), this heterogeneity could not be solved by exclusion of one study (Figure 14).

**Figure 15:**
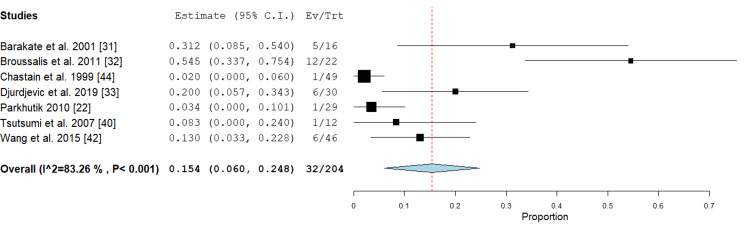
Forest plot for the analysis of retreatment

Retreatment: This outcome was mentioned in the PTAS subgroup only by seven studies [22,31-33,40,42,44] with an incidence rate of 15.4% (95% CI {6%, 24.8%}). Pooled analysis was heterogeneous (P < 0.001; I² =83.26 %); this heterogeneity could not be solved by excluding one study, as shown in Figure 15.

**Figure 16:**
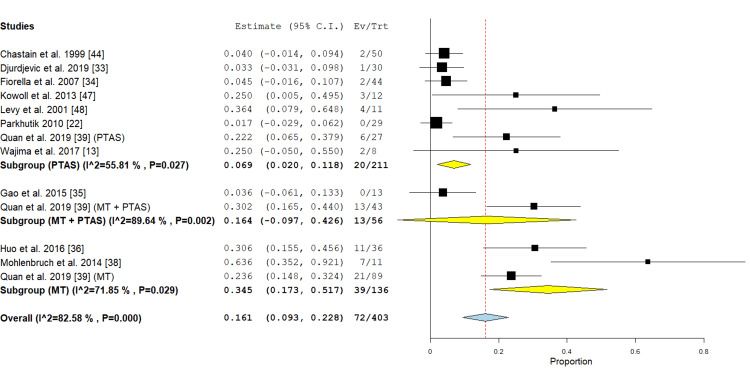
Forest plot for the analysis of 90 days mortality PTAS: percutaneous transluminal angioplasty and stenting, MT: mechanical thrombectomy.

Ninety days mortality: In the PTAS subgroup, eight studies reported this outcome [22,33,34,39,44,47,48,50] with an incidence of 6.9% (95% CI {2%, 11.8%}) the analysis showed moderate heterogeneity (P 0.027; I² = 55.81 %). In the MT subgroup three studies were included in the analysis [36,38,39] with an incidence of 34.5% (95% CI {17.3%, 51.7%}), the analysis was heterogeneous (P=0.029; I² = 71.85 %). In the PTAS+MT subgroup two studies reported this outcome [35,39] with an incidence rate of 16.4% (95% CI {-0.097, 0.426}), the analysis was heterogeneous (P= 0.002; I² = 89.64 %) (Figure 16).

Discussion

Our analysis found that the use of mechanical thrombectomy alone is associated with the highest rates of adverse events and mortality. Percutaneous transluminal angioplasty with or without stenting is the most effective and least associated with mortality. Additionally, we found that the intervention led to a significant increase in NIHSS score but did not significantly increase the MRS score at discharge.

Endovascular therapy includes balloon-mounted stents, balloon angioplasty alone, and self-expandable stents with or without prior angioplasty [51]. Zhang et al. found that treatment with self-expanding stents has a higher risk of restenosis and longer operative time than treatment with balloon-mounted stents in patients with symptomatic intracranial vertebrobasilar arterial stenosis [50].

A review by Luo et al. suggested endovascular treatment as an effective and safe option for the management of intracranial atherosclerotic stenosis if it is used in selected patients and performed with an experienced team who could carefully manage the patients before, during, and after the procedure [51]. These results appear inconsistent with our results. Nevertheless, Goyal et al., in their review, presented challenges with the implementation of endovascular therapy that needed to be resolved. The first, viable implementation of the outcomes across a large number of people; the second, observing, empowering, and approving the new treatments that bring about additional improvements; and third, making a framework to permit induction of outcomes of trials on patients that were not previously tested. Finally, increasing the accessibility of endovascular therapy in developing countries [52]. 

Thrombolysis, either intravenous thrombolysis or local intra-arterial thrombolysis are among the treatments used to manage vertebrobasilar system stenosis or occlusion. They are considered the most treatment used for revascularization of acute vertebrobasilar artery occlusion. Intra-arterial thrombolysis (IAT) achieves a higher rate of revascularization than intravenous thrombolysis (IVT), but there is not much difference between the efficacy of both [53,54]. Moreover, there is a strong relationship between the occlusion site and the efficacy of intravenous thrombolysis to achieve revascularization and earlier neurological recovery successfully. The chance for successful revascularization is least with terminal internal carotid artery occlusion, but a higher chance for revascularization is with smaller and more distal occlusion [55,56]. Lindeberg et al. revealed that the recanalization rate is higher in patients treated with intravenous thrombolysis than those treated with endovascular techniques [53].

Endarterectomy and reconstruction are surgical treatments offered for the management of atherosclerotic stenosis of the vertebral artery. However, their performance has been diminished in recent years and replaced with endovascular interventions for refractory cases to medical treatment [57].

Our analysis's main strength is the inclusion of a large number of studies performed in different countries and the absence of any evidence of heterogeneity in the analysis. Conversely, cohort design, either retrospective or prospective with a moderate to high risk of bias, is considered the main limitation. We used subgroup analysis to overcome the inconsistency among the included studies. Further studies are recommended to compare the efficacy and safety of the medical treatment and endovascular therapy in managing vertebrobasilar system stenosis or occlusion.

## Conclusions

With regard to VBAO, we conclude that PTA with or without stenting is associated with better outcomes and a lower rate of mortality when compared to MT alone.
